# Use of Long-Acting Injectable Antipsychotics in a Clinical Sample of Community-Dwelling Patients with Schizophrenia-Spectrum Disorders in Rural Greece

**DOI:** 10.3390/jcm12072508

**Published:** 2023-03-26

**Authors:** Vaios Peritogiannis, Fotini Tsoli, Panagiota Gioti, Maria Bakola, Eleni Jelastopulu

**Affiliations:** 1 Mobile Mental Health Unit of the Prefectures of Ioannina and Thesprotia, Society for the Promotion of Mental Health in Epirus, 45445 Ioannina, Greece; 2 Department of Public Health, School of Medicine, University of Patras, 26500 Patras, Greece

**Keywords:** antipsychotics, community mental healthcare services, long-acting injectables, mobile mental health units, rural areas, schizophrenia-spectrum disorders, treatment adherence

## Abstract

Data on the use of long-acting injectable antipsychotics (LAIs) in rural community mental healthcare settings are scarce. This study aimed to investigate the prescription patterns of LAIs in a clinical sample of patients with schizophrenia-spectrum disorders in rural Greece. All patients with schizophrenia-spectrum disorders who regularly attend the Mobile Mental Health Unit of the prefectures of Ioannina and Thesprotia (MMHU I-T) in northwestern Greece were included in the study. The sample consists of 87 patients (59 males and 28 females) with a mean age of 54.4 years and a mean illness duration of 28 years. Most patients (72.4%) received antipsychotic monotherapy, and nearly 30% received an LAI formulation, mostly a second-generation LAI (20 of 26 patients, 76.9%). The treatment regimen comprised benzodiazepines in one-third of the patients and antidepressants in one-quarter. There was no statistically significant association between treatment regimen and the clinical and demographic variables studied, except for biological sex (female). The percentage of patients treated with LAIs in this study was almost three times higher than the rate previously reported in Greece and is higher than the rates reported in other countries. Patients with schizophrenia-spectrum disorders in rural Greece may have adequate access to innovative treatment with second-generation LAIs. Further research is needed to demonstrate the cost-effectiveness of LAI treatment in rural communities and to elucidate the factors associated with such treatment.

## 1. Introduction

Schizophrenia and related psychoses (the so-called psychotic disorders) are chronic and severe mental disorders with modest prognosis and often poor long-term outcome [1], and they can lead to disability in a substantial proportion of patients [2]. These disorders often require long-term or indefinite treatment with antipsychotic medications that are effective in eliminating psychotic symptoms and are usually well tolerated by patients [3]. The efficacy of antipsychotics in the short-, mid-, and long-term treatment of psychotic disorders has been consistently supported by evidence [4]. Moreover, maintenance antipsychotic treatment has been associated with lower mortality in people with schizophrenia when compared to no antipsychotic treatment [5]. Metabolic disturbance and tardive dyskinesia may be the most troublesome adverse effects of chronic antipsychotic use [5]. In cases of treatment-resistant schizophrenia, the use of clozapine is recommended by all relevant clinical practice guidelines [6]. It has been shown that clozapine may have superior effects on all symptoms of schizophrenia, compared to other antipsychotics, and may also reduce relapse rates in treatment-resistant cases [7]. Importantly, despite its well-known metabolic adverse events, clozapine use has been associated with lower mortality rates, hospitalization rates, and all-cause discontinuation rates [7]. 

However, treatment of psychotic disorders may be frequently undermined by patients’ non-adherence to medication and disengagement from mental health services [8,9,10]. Non-adherence is frequent across all domains of medicine, but rates may be particularly high in patients with severe mental illness [11]. Antipsychotic treatment adherence rates have been reported to be as low as 40–60%, and it has been suggested that poor treatment adherence is associated with poor outcomes in schizophrenia [12]. Factors that have been consistently associated with non-adherence include lack of insight, the direct impact of symptoms (such as depression, cognitive impairment, and positive and negative symptoms), social isolation, comorbid substance misuse, stigma, beliefs about treatment risks and benefits, and the fragmentation of mental health services in some countries [11,13]. Factors positively related to adherence include a good therapeutic relationship with the treating physician and perception of benefits of medication [14]. Treatment non-adherence may have several adverse consequences, such as high risk of relapse, hospitalizations, and suicide [14]. Accordingly, several measures of adherence behaviors have been employed, though with important limitations, and various interventions to improve adherence have been studied. Current evidence-based interventions to improve adherence include family therapy, technology-based interventions, and strategies combining depot medication with psychoeducation [15].

Long-acting injectable antipsychotics (LAIs) have been shown to be a reliable method to monitor patients’ treatment adherence. Over the last few decades, the introduction of second-generation or atypical LAIs for the maintenance treatment of schizophrenia has yielded favorable results in terms of relapse and hospitalization prevention, as well as patient tolerability and acceptability [16]. There is some evidence that LAIs have therapeutic advantages over oral antipsychotics that are not only related to improved treatment adherence. A recent meta-analysis of randomized, cohort, and pre-post studies comparing LAIs with oral antipsychotics found that LAIs were consistently associated with significantly lower risk of relapse and/or hospitalization and better outcomes [17]. Most importantly, it has been suggested that the benefits observed in clinical trials may be even greater in naturalistic studies and real-world settings [18,19]. Moreover, a previous analysis of mortality in a nationwide cohort of 29,823 patients with schizophrenia in Finland reported the lowest mortality rates with second-generation LAIs and an overall 33% lower risk of death during treatment with LAI compared with equivalent oral treatment [20]. More recently, a large study in Taiwan on newly diagnosed patients with schizophrenia found that use of LAIs was associated with decreased all-cause mortality and suicide risk in patients [21]. 

In addition to being used for the treatment of schizophrenia, LAIs have been also used in the treatment of delusional disorder. Although these two syndromes share similar psychotic features, there are many differences between them. Patients with delusional disorder are more likely to have comorbid substance abuse, later age at illness onset, more affective symptoms, greater lack of insight, poorer response to antipsychotic medication, and better occupational and social functioning, compared to those with schizophrenia. Delusions in delusional disorder may be fewer but more severe, whereas conviction of delusional experience is higher in those patients [22,23]. Moreover, there may be gender differences with regard to delusional themes, depression and anxiety comorbidity (more common in women), and substance use disorders (more common in men) that could be attributable to sociocultural factors. Interestingly, menopause may influence symptom expression and comorbidities in women [24]. A recent observational Swedish registry study found that treatment with antipsychotics was associated with a reduced risk of hospitalization due to psychosis and work disability in delusional disorder and that LAI or clozapine treatment was the most effective treatment in this regard [25].

Despite the available evidence on the benefits of LAIs in the treatment of schizophrenia and the respective clinical recommendations concerning their use, they appear to be under-prescribed in routine clinical practice, although rates may vary by country and setting. A previous community-based study in France examining the initiation of LAI treatment under naturalistic conditions reported that LAI use was consistently less than 10% over an eight-year period [26], whereas the corresponding rate in the United States was slightly higher (13%) [27]. Another study in Italy, which looked at patients attending community mental health centers in the Province of Verona, found that the annual frequency of new treatments with LAIs was relatively stable over the five-year study period. On average, 5.4% of patients treated with antipsychotics were prescribed LAIs [28]. In Spain, the percentage of second-generation LAI prescriptions compared to total second-generation antipsychotic prescriptions increased from 9.8% to 16.4% over a six-year period (2011–2016) [29]. Very recently, a study on inpatients with schizophrenia-spectrum disorders in Switzerland found a 13.9% prescription rate for LAIs [30]. 

Less is known about the use of LAIs in rural community mental healthcare settings. There is some evidence that LAIs in those settings can improve treatment adherence [31] and that such treatment can be cost-effective [32]. Since schizophrenia is a disease with a multifactorial etiology in which social factors play a key role and treatment adherence is a challenge to its therapy, it is important to consider treatment in multiple cultures, milieus, and countries. Therefore, the aims of the present study were to investigate the prescription patterns of LAIs in patients with psychotic disorders attending a community-based mental healthcare service in rural Greece and to investigate clinical and demographic variables that may be associated with the use of LAIs in this patient population. The main hypothesis of the study was that prescription of LAIs would be correlated with clinical and perhaps demographic characteristics of patients. 

## 2. Materials and Methods

### 2.1. The Treatment Setting

In rural and remote areas in Greece, mental healthcare in the community is primarily provided by locally based Mobile Mental Health Units (MMHUs) [33,34]. These interdisciplinary teams offer a wide range of evidence-based community interventions for patients with mental disorders, with a focus on severely ill patients, including those with a schizophrenia-spectrum disorder [35]. The MMHU in the prefectures of Ioannina and Thesprotia (MMHU I-T) serves a population of approximately 100,000 inhabitants in rural areas of the Epirus region in northwestern Greece. Priority is given to patients with psychotic disorders, and the MMHU I-T places particular emphasis on treatment engagement and monitoring of antipsychotic drug treatment [36,37]. This study was approved by the Institutional Review Board of the Society for the Promotion of Mental Health in Epirus (Δ.2/5-12-2022), and the need for patients’ informed consent was waived, as it is a non-interventional survey that relied on clinical records.

### 2.2. Patient Sample

This is a cross-sectional study on patients with schizophrenia-spectrum disorders (F20–F29, according to the International Classification of Diseases, Tenth Revision (ICD-10)). All active patients who attended the MMHU I-T and the hybrid Assertive Community Treatment team [38] during the data acquisition period (August 2022) were included. Patients were rated as active when they regularly attended scheduled follow-up appointments. These patients, despite some fluctuations in their symptomatology, are considered “stabilized”, do not require hospitalization, and live in the community with varying levels of functioning. Patients were excluded if they received medication for <3 months prior to the study period, and if their age was <18 years. Clinical (illness duration, hospitalizations, follow-up duration, history of alcohol/substance abuse, treatment regimen) and demographic (age, biological sex, carer) information was retrieved from the clinical records of the patients. The patients’ histories of alcohol/substance abuse were recorded based on their own and other informants’ reports.

### 2.3. Statistical Analysis

We performed statistical analysis using SPSS version 25.0 (SPSS Inc., Chicago, IL, USA). Continuous variables were expressed as mean ± standard deviation (M ± SD), while categorical variables were expressed as absolute numbers (n) and percentages (%). The normality of the variables was tested with the Shapiro-Wilk test and the normal Q-Q plot, the detrended normal Q-Q plot, and the box plot. For all tests, statistical differences were determined to be significant at *p* < 0.05. We used the independent *t*-test when comparing the means of two groups. The chi-squared test and Fisher’s exact test were used to determine if the proportions for one categorical variable differed from the values of the other categorical variable.

## 3. Results

A total of 87 patients with schizophrenia-spectrum disorders, including 59 men and 28 women, are currently undergoing treatment with the MMHU I-T and receiving antipsychotics. The patients’ demographic and clinical characteristics are presented in Table 1. With regard to diagnosis, most of the included patients suffer from schizophrenia (n = 68, 78.2%), and the other included diagnoses are delusional disorder (n = 7, 8%), schizoaffective disorder (n = 7, 8%) and other psychoses (n = 5, 5.8%).

The sample is composed of middle-aged, chronically ill patients with schizophrenia-spectrum disorders, with a mean age of 54.4 years (±12.1) and a mean illness duration of 28 years (±14.4). These patients have been followed by the MMHU I-T for an average of 7 years (±4.9). Regarding the patients’ medication, the vast majority (72.4%) received antipsychotic monotherapy, whereas nearly 30% of the patients received an LAI formulation, mostly a second-generation LAI (20 out of 26 patients, 76.9%). Details on the employment of specific antipsychotics in each group of patients are presented in Table 2. The treatment regimen also comprised benzodiazepines for one-third of patients and antidepressants for nearly one-quarter of patients. 

We conducted an independent samples *t*-test to examine potential differences in age, illness duration, total number of hospitalizations, and follow-up duration between patients receiving an LAI formulation and those receiving oral antipsychotic treatment. There was no statistically significant difference in the aforementioned variables between the two groups of patients. For the estimation of potential correlations between antipsychotic treatment formulation (oral vs. LAI) and variables such as biological sex, treatment regimen (antipsychotic monotherapy vs. antipsychotic combination), concomitant benzodiazepine treatment, and concomitant antidepressant treatment, a chi-square test for association was conducted. All expected cell frequencies were greater than five. Our analysis did not show any statistically significant association between treatment formulation and the examined variables, with the exception of biological sex (Table 3). 

**Figure 1:**
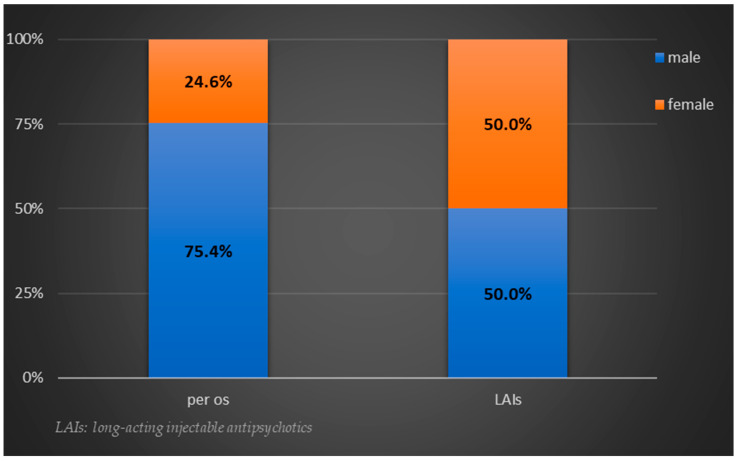
Percentage distribution of biological sex per group of antipsychotic formulation.

Based on the results of the chi-square test for association, there was a statistically significant difference in the proportion of women and men receiving an LAI formulation (χ^2^(1) = 5.393, *p* = 0.020). Specifically, a higher percentage of women received LAIs compared to men (Figure 1).

## 4. Discussion

This is the first Greek study that addresses treatment with LAIs in a clinical sample of patients with schizophrenia-spectrum disorders residing in rural areas. The percentage of patients who received treatment with LAIs was almost 30%. Compared with previous data from our country that involved various treatment settings [39], this rate is a nearly three-fold increase. However, previous data on rates of treatment with LAI in Greece were published 20 years ago, when only first-generation antipsychotics were available in LAI formulations. Present data mostly involve the administration of second-generation LAIs (20 out of 26 patients, 76.9%). Presumably, the more benign side-effect profile of these agents makes clinicians less reluctant to prescribe second-generation LAIs, and patients more willing to accept this treatment. Notably, other previous research in Greece reported an even lower rate of LAI prescription (5.5%), mostly involving the use of risperidone [40]. Perhaps the treatment regimen of bi-monthly injections may not be popular among patients or clinicians, and this may account for the low rate. 

The present sample comprises chronically ill (mean illness duration 28 years), mostly middle-aged (mean age 54.4 years) patients. Those patients usually live with elderly parents who may not be able to monitor their adherence with oral medication. Indeed, more patients of the present sample would be eligible for treatment with LAIs, but on several occasions, patients were reluctant or even resistant to engage in such a treatment. In a recent study of inpatients in Switzerland, it was found that only 28.1% of the patients treated with an antipsychotic that is available as an LAI formulation were prescribed an LAI, suggesting that only a portion of patients who may be eligible for LAI treatment actually receive it [30]. Moreover, in a study in Italy on community-dwelling patients with psychotic disorders who were prescribed an LAI, only 20.7% steadily continued that treatment over the six-month follow-up [28], which indicates the challenges in maintaining such treatment.

This study involved rural patients who are treated in a community mental health service. It is not known what the percentage of treatment with LAIs is in patients who attend other public mental health services. Moreover, there are no data available regarding patients receiving treatment in the private practice setting. A large portion of patients with schizophrenia-spectrum disorders in Greece are treated by private practice psychiatrists [41], but research in those settings is limited. Comparisons with other patients who attend other services in other locations were not made. However, it has been previously reported that place of residence may impact pharmacological treatment of schizophrenia and related disorders due to limited resources and mental health service shortages [42,43]. Distance and the rural setting may not account for the treatment with LAIs in the present study, because patients were regularly followed up with by the locally based MMHU and received comprehensive mental healthcare. On the other hand, the decision to prescribe LAIs may be affected by several factors, including the physician’s familiarity with LAIs, the patient’s preference, and the availability of the formulation [44]. Such factors could probably account for the wide variability in LAI use across settings. 

There were no differences between the two groups (oral vs. LAI treatment) in terms of age, disease duration, and follow-up duration. Other parameters, such as the impact of caregiving status, could not be assessed due to the relatively small study sample. The number of hospitalizations did not differ statistically between the two groups of patients. It has been previously shown that treatment delivered by the MMHUs in rural Greece may reduce the number of hospitalizations, both voluntary and involuntary, as well as the length of hospital stay in patients with severe mental illness [45,46].

Among the clinical and demographic variables that have been examined in the present study, only female gender is statistically significantly associated with LAI use. This does not necessarily mean that female patients in this study have a more positive attitude toward LAIs or that they are more frequently eligible for such treatment, but another interpretation could be that women would be more easily forced by caregivers to accept such treatment to address poor adherence. There is evidence that female patients with schizophrenia are commonly amenable to domestic coercion and control [47,48], and this could be the case with the female patients in the present sample. This finding is clinically relevant, as recent research has suggested that female gender may pose a higher risk of antipsychotic treatment non-adherence [10]. However, in a very recent study of an inpatient ward in Switzerland, male gender was associated with LAI prescription [30]. Additionally, this finding may have clinical implications. It has been previously shown that in the region investigated in our study, male gender is mostly associated with involuntary admission status [49] and with the subsequent application of coercive physical measures during hospitalization [50]. Subsequently, several of those admissions and their adverse consequences would have been avoided if more male patients had been prescribed an LAI regimen. Accordingly, interventions for male patients are needed if we are to increase LAI use in those patients. 

There were no differences between the two groups with regard to the rates of antipsychotic co-medication, nor with regard to co-administration of benzodiazepines or antidepressants. Unlike other recent reports from community mental health settings [28,51], in the present study, most patients on LAIs (17 out of 26, 65.4%) were in antipsychotic monotherapy. Supposedly, the practice of combining LAIs with oral medication corresponds to the complexity of cases, which may require additional drug treatment; the patients’ co-morbidities (e.g., depressive symptoms); and the different diagnoses that are included in the F20–F29 cluster of the ICD-10. This was not the case in the sample of patients in the present study. This observation may not necessarily mean that those patients did not need concomitant oral medication; it could also be interpreted as an indication of the patients’ reluctance or unwillingness to receive any additional oral medication. With regard to the co-administration of benzodiazepines, it has been previously suggested that such treatment may be initially employed for the short-term management of agitation or insomnia in those patients and also may become part of their regular treatment regimen afterwards [52].

Previous research in Greek acute psychiatric settings has shown that a large proportion of patients maintain adequate decision-making capacity with regard to their treatment [53,54]. On the other hand, research on patients receiving LAIs has found that over half of the participants showed poor understanding of the information given regarding their illness and its treatment [55]. This may mean that clinicians preserve this treatment regimen for patients who are unable to adequately process the information regarding their treatment and thus may be at risk for treatment non-adherence. This may particularly be the case of patients with delusional disorder, who often have poor insight. Treatment with an LAI would be preferable for those patients, given the advantages of LAIs in this population [25]. Such treatment is relevant for these patients because, unlike patients with schizophrenia, many patients with delusional disorder may indeed work, and treatment could help them maintain employment [56].

There are some concerns regarding the prescribing of LAIs during the COVID-19 pandemic. Some studies have suggested that there may be a reduction in LAI initiation. A recent study in Romania found a 48.3% reduction in new LAI prescriptions compared to the pre-pandemic period [57], whereas in the US, LAI prescribing remained unchanged in 64.6% of cases, according to a survey among 401 LAI prescribers [58]. However, other research yielded different results. An observational study in Italy in a real-world clinical setting found no significant differences between 2020 and 2019 in the total number of patients on LAI treatment and the number of dropouts, although a significant reduction in new LAI starts was observed [59]. Another retrospective observational study in Canada examined national and provincial patient-level longitudinal prescribing data from pharmacies’ database that corresponded to 72% of national prescriptions. No impact of the COVID-19 pandemic on the LAI prescription rate at the national or provincial level was found. That is, rates of LAI initiations and discontinuations were not significantly different prior to and during the pandemic [60]. Similarly, in Greece, LAI administration was not changed during the pandemic, as the number of visits to a depot clinic remained the same as during the pre-pandemic period [61]. In the case of MMHUs, the ease of patients’ access to the service is associated with high rates of treatment engagement [62] and may enable the application of injectable treatment.

### Limitations and Strengths

The present study has some limitations. The relatively small patient sample did not allow for further statistical analysis, and some information may have been missed. It is not known whether the results of the present study can be generalized to similar settings. Recent research has suggested that there may be noticeable differences among MMHUs in rural Greece [63]. However, it is possible that the global trend to prescribe LAIs for patients with a history of poor treatment adherence [44] may also be the case across MMHUs in rural Greece.

The objective of the present study was to explore the use of LAIs in patients with psychotic disorders attending community mental health services in rural areas. Data on patients’ symptomatology, functioning, or other aspects of outcome are not presented since the cross-sectional design of the study would preclude any conclusions from being drawn regarding the association of medication with outcome. However, it is worth noting that a large proportion of patients with psychotic disorders that attend the MMHUs have been previously reported to have favorable long-term outcomes [64,65]. Future prospective research should address the impact of LAI treatment on patients’ outcomes in rural settings. In particular, it would be interesting to assess clinical outcomes like mortality, suicide attempts, and psychotic relapses compared to patients receiving oral antipsychotics. 

It is worth noting that the prescription of LAIs in our sample does not correspond solely to the prescribing practices of the psychiatrists of the MMHU I-T. Rather, some patients received an LAI formulation before their referral, sometimes during their hospitalization. From a clinical perspective, it is important to note that patients with such severe and chronic mental disorders in rural areas seem to have adequate access to innovative treatments, that is, second-generation LAIs that could enable treatment adherence. This is probably explained by the global insurance coverage in Greece and the interventions by the locally based MMHUs. The use of LAIs in the rural community setting may be even more relevant because a previous systematic review suggested that LAIs reduce the risk of relapse when compared with oral antipsychotics in outpatients with schizophrenia when combined with quality psychosocial interventions [18], which is the case with MMHUs in rural Greece [66]. Further research is warranted to establish whether the use of LAIs in rural community mental healthcare settings enables treatment adherence and to explore the cost-effectiveness of such treatment.

## 5. Conclusions

Rates of the LAI use in patients with psychotic disorders in rural Greece seem to be higher than previously reported in other settings in Greece and elsewhere. Patients in those underserved areas have access to innovative treatment with second generation LAIs, which may enable treatment adherence. Women seem to be prescribed such treatment regimens more frequently, and this may have clinical implications and should guide interventions toward the increase of LAI prescription in men. More research is needed with multi-center studies to demonstrate the cost-effectiveness of LAI treatment in rural community settings in Greece and to elucidate the clinical and demographic factors associated with such treatment. The attitudes of patients and clinicians toward LAI should also be studied.

## Figures and Tables

**Table 1:** Patients’ demographic and clinical characteristics.

Age (years, mean ± SD)		54.4 ± 12.1	
Illness duration (years, mean ± SD)		28.0 ± 14.4	
Hospitalizations (mean ± SD)		3.4 ± 4.5	
Follow-up duration (years, mean ± SD)		7.0 ± 4.9	
		n	%
Antipsychotic medication	Oral	61	70.1
	LAI	26	29.9
Biological sex	Female	28	32.2
	Male	59	67.8
Treatment regimen	Monotherapy	63	72.4
	Combination	24	27.6
History of alcohol/substance abuse	No	61	70.1
	Yes	26	29.9
Carer	No	19	21.8
	Yes	54	62.1
	Family with other patients with SMI	14	16.1
Benzodiazepines	No	58	66.7
	Yes	29	33.3
Antidepressants	No	65	74.7
	Yes	22	25.3

LAI: long-acting injectables; SMI: severe mental illness.

**Table 2:** Patients’ antipsychotic regimen.

Generic Drug Name	Oral Treatment	LAI Formulation
Olanzapine	20	5
Risperidone	16	2
Paliperidone	1	13
Aripiprazole	12	-
Quetiapine	12	NA
Amisulpride	8	NA
Asenapine	1	NA
Haloperidol	6	4
Chlorpromazine	1	NA
Zuclopenthixol	-	2
Trifluoperazine	1	NA
Clozapine	7	NA

NA: not available.

**Table 3:** Comparisons of patients on oral antipsychotic treatment and on LAI treatment.

	Patients on Oral Antipsychotics (n = 61)	Patients on LAI (n = 26)	Statistical Test	*p*
Age (years, mean, SD)	54.3 (12.9)	54.6 (10.4)	t(85) = −0.112	NSS
Biological sex (male/female)	46/15	13/13	χ^2^(1) = 5.393	0.02
Illness duration (years, mean, SD)	27.6 (15)	28.8 (13.8)	t(85) = −0.338	NSS
Hospitalizations (mean, SD)	3.7 (5.2)	2.7 (2.2)	t(85) = 0.975	NSS
History of alcohol/substance abuse	17	9	χ^2^(1) = 0.396	NSS
Follow-up duration (years, mean, SD)	7.1 (4.7)	6.8 (5.5)	t(85) = 0.260,	NSS
Monotherapy vs. antipsychotic combination	46 vs. 15	17 vs. 9	χ^2^(1) = 0.917	NSS
Concomitant benzodiazepine	24	5	χ^2^(1) = 3.319	NSS
Concomitant antidepressant	19	3	χ^2^(1) = 3.710	NSS

NSS: not statistically significant.

## Data Availability

Data are kept in the patients’ electronic charts of the Mobile Mental Health Unit of the prefectures of Ioannina and Thesprotia and are confidential.
